# Change analysis for intermediate disease markers in nutritional epidemiology: a causal inference perspective

**DOI:** 10.1186/s12874-024-02167-9

**Published:** 2024-02-27

**Authors:** Dan Tang, Yifan Hu, Ning Zhang, Xiong Xiao, Xing Zhao

**Affiliations:** 1 West China School of Public Health and West China Fourth Hospital, Sichuan University, Chengdu, China; 2 Xiamen Center for Disease Control and Prevention, Xiamen, China

**Keywords:** Intermediate disease marker, Concurrent change-change analysis, Change-score analysis, Lagged change-change analysis, Causal inference, Unobserved time-invariant heterogeneity, Simulation study

## Abstract

**Background:**

Several approaches are commonly used to estimate the effect of diet on changes of various intermediate disease markers in prospective studies, including “change-score analysis”, “concurrent change-change analysis” and “lagged change-change analysis”. Although empirical evidence suggests that concurrent change-change analysis is most robust, consistent, and biologically plausible, in-depth dissection and comparison of these approaches from a causal inference perspective is lacking. We intend to explicitly elucidate and compare the underlying causal model, causal estimand and interpretation of these approaches, intuitively illustrate it with directed acyclic graph (DAG), and further clarify strengths and limitations of the recommended concurrent change-change analysis through simulations.

**Methods:**

Causal model and DAG are deployed to clarify the causal estimand and interpretation of each approach theoretically. Monte Carlo simulation is used to explore the performance of distinct approaches under different extents of time-invariant heterogeneity and the performance of concurrent change-change analysis when its causal identification assumptions are violated.

**Results:**

Concurrent change-change analysis targets the contemporaneous effect of exposure on outcome (measured at the same survey wave), which is more relevant and plausible in studying the associations of diet and intermediate biomarkers in prospective studies, while change-score analysis and lagged change-change analysis target the effect of exposure on outcome after one-period timespan (typically several years). Concurrent change-change analysis always yields unbiased estimates even with severe unobserved time-invariant confounding, while the other two approaches are always biased even without time-invariant heterogeneity. However, concurrent change-change analysis produces almost linearly increasing estimation bias with violation of its causal identification assumptions becoming more serious.

**Conclusions:**

Concurrent change-change analysis might be the most superior method in studying the diet and intermediate biomarkers in prospective studies, which targets the most plausible estimand and circumvents the bias from unobserved individual heterogeneity. Importantly, careful examination of the vital identification assumptions behind it should be underscored before applying this promising method.

**Supplementary Information:**

The online version contains supplementary material available at 10.1186/s12874-024-02167-9.

## Background

In the past decades, it has become increasingly prevalent to study sensitive disease biomarkers or intermediate endpoints of diseases, such as weight gain, blood pressure, glycemia, lipid profiles, and other cardiometabolic or inflammation-related biomarkers in epidemiology, which can help to identify disease risk factors earlier and provide potential pathways linking these factors to distal diseases [1–4]. In contrast to dichotomous disease status, intermediate biomarkers are continuous indicators, which are more sensitive to various exposure factors and tend to fluctuate over short periods of time as exposure changes [2, 3]. The longitudinal cohort data with repeated measurements could capture the covariation relationships between exposures, confounders, and outcome indicators over time, providing an ideal data structure for clarifying the causal associations between time-varying exposures of interest and intermediate biomarkers.

In practice, while prospective studies on the relationship between diet (including other lifestyle factors such as physical activity) and pre-disease intermediate biomarkers are tremendous, the analytical methods are various, which have produced very different even contradictory results [5–7]. Commonly used approaches mainly fall into the following three categories. The first approach is change-score analysis, which involves modeling the association of baseline exposure and subsequent biomarker change [7–9]. The second is concurrent change-change analysis, which evaluates the association of exposure change and biomarker change within the same timespan [7, 10–15]. The last is lagged change-change analysis, which models the association of previous exposure change and subsequent biomarker change [5–7, 12]. An empirical comparison study has thoroughly evaluated and compared these three approaches based on three famous large-scale prospective cohorts [7]. The results showed that concurrent change-change analysis could produce the most robust, consistent, and biologically plausible estimates and therefore was a superior and recommended analytical method to assess the relationship of diet with weight gain in prospective cohort studies [7]. Since then, this method has been widely used in longitudinal studies to explore whether and to what extent the change in diet leads to parallel change in weight (or other adiposity measures: BMI, waist circumference) [16–21] as well as many cardiometabolic and inflammation-related biomarkers [22–24] in a relatively short time.

Although such concurrent change-change analysis is appealing in epidemiology and empirical evidence suggests that it outperforms other analysis methods, few studies explicitly elucidate and compare the rationale of the above three approaches from the perspective of causal inference, including the underlying causal model, causal effect estimand, appropriate causal interpretation, etc. Therefore, this article intends to understand and compare these methods under the framework of causal inference, intuitively illustrate it with directed acyclic graph (DAG), further clarify the strengths and limitations of the recommended concurrent change-change analysis through simulations, and thereby have a better understanding of why concurrent change-change analysis usually works while others do not, and under what circumstance it should work.

## Theoretical interpretations

### The underlying causal model and estimand

#### Concurrent change-change analysis

The intuitive idea of concurrent change-change analysis is to capture the covariation pattern of exposure and outcome, then answer the question of whether and how changes in exposure cause changes in disease markers. This approach only models the within-individual variation thus could remove the influence of between-individual heterogeneity once the model assumptions are satisfied. In fact, the concurrent change-change analysis method is identical to the fixed effects model (FEM) developed in the econometric literature. FEM is a classical causal inference method commonly used in repeated measures data in econometrics and sociology, which is based on the principle of self-control [25, 26]. The typical linear causal model for two-way FEM is:

1 $${y}_{it}=\beta {x}_{it}+\gamma {z}_{it}+{u}_{i}+{\lambda }_{t}+{\epsilon }_{it}$$

Here, to aid the understanding of this causal model, we used the illustration example of evaluating the effect of dairy intake on weight in a prospective cohort study. Thus, ${y}_{it}$ is the weight of individual $i$ measured at time $t$, ${x}_{it}$ is the collected dairy intake of this individual at time $t$, ${z}_{it}$ is some other observed time-varying covariates (e.g. intake of other food, physical activity, sleep status, and so on), ${u}_{i}$ denotes the effect of unobserved individual-specific characteristics (such as genetic predisposition) and ${\lambda }_{t}$ represents the time-specific effects (reflects the effects of unobserved time-varying variables, such as economic growth, health literacy and so on), ${\epsilon }_{it}$ is the random error term. It is worth noting that FEM assumes a concurrent influence of exposure on outcome indicator (${x}_{it}\to {y}_{it}$), that is, the targeted estimand of FEM is the effect of exposure on contemporaneously measured outcome.

Suppose we have only two-wave $t=0, 1$ panel data of $i=1,\cdots ,N$ individuals, we can obtain two equations according to model (1):

$${y}_{i0}=\beta {x}_{i0}+\gamma {z}_{i0}+{u}_{i}+{\lambda }_{0}+{\epsilon }_{i0}$$

$${y}_{i1}=\beta {x}_{i1}+\gamma {z}_{i1}+{u}_{i}+{\lambda }_{1}+{\epsilon }_{i1}$$

Differencing above two equations can wipe out the time-invariant unobserved term ${u}_{i}$:

2 $${\varDelta y}_{i}={(\lambda }_{1}-{\lambda }_{0})+\beta {\varDelta x}_{i}+\gamma {\varDelta z}_{i}+{\varDelta \epsilon }_{i}=\alpha +\beta {\varDelta x}_{i}+\gamma {\varDelta z}_{i}+{\epsilon }_{i}^{*}$$

The model (2) is a typical analytical model used in concurrent change-change analysis. Therefore, the estimand of concurrent change-change analysis is that of FEM ($\beta :{x}_{it}\to {y}_{it}$), which is the average causal effect of dairy intake on contemporaneously measured weight in this example. The most appealing strength of this method is that it takes advantage of the idea of self-control, which makes it rely only on intra-individual variation, and the time-invariant unobserved heterogeneity (${u}_{i}$, such as the heterogeneous genetic background) is eliminated by differencing (other “within transformations” such as demeaning could also eliminate the term). Such unmeasured time-invariant confounding is prone to most observational studies in which effect estimations usually rely both on variations within and between individuals.

However, there were several vital causal identification assumptions for application of FEM [26, 27]:

(i) The strict exogeneity (SE) assumption of the error term: for each $i=1,\cdots ,N$ and $t=0,\cdots , T$,

$$E\left[{\epsilon }_{it}|{\varvec{X}}_{\varvec{i}},{\varvec{Z}}_{\varvec{i}},{\varvec{\lambda }}_{\varvec{t}},{u}_{i}\right]=0$$

${\varvec{X}}_{\varvec{i}}$ and ${\varvec{Z}}_{\varvec{i}}$ is a $T\times 1$ vector of exposure variables or covariates for unit $i$, respectively. ${\varvec{\lambda }}_{\varvec{t}}$ is a $T\times 1$ vector of time-specific effect terms. This assumption forbids the correlation of current error ${\epsilon }_{it}$ with past, present, and future values of regressors, which implies the absence of dynamic causal relationships between exposure and outcome variables across different periods, specifically including the causal relation of the past outcome ${y}_{i,t-1}$ and current outcome ${y}_{it}$ (autocorrelation), the causal relation of past exposure ${x}_{i,t-1}$ and current outcome ${y}_{it}$ (lag effects), or the causal relation of past outcome ${y}_{i,t-1}$ and current exposure ${x}_{it}$ (reverse causation) [27].

(ii) The common trend (CT) assumption for different individuals: for each $t=0,\cdots , T$ and each possible exposure level $x$,

$$E\left[{y}_{it}^{x}-{y}_{i0}^{x}|{z}_{i0},{z}_{it}\right] \text{i}\text{s}\ \text{c}\text{o}\text{n}\text{s}\text{t}\text{a}\text{n}\text{t}\ \text{f}\text{o}\text{r}\ i=1,\cdots ,N$$

${y}_{it}^{x}$ denotes the potential counterfactual outcome of individual $i$ at time $t$ if the exposure is at $x$ level, this assumption requires individual outcome trajectories parallel to each other had they not changed their exposure level, which implies that the time-specific effects (${\lambda }_{t}$) are constant (or equivalently, unmeasured time-varying variables are identical) among individuals after conditioning on all the measured confounders.

#### Change-score analysis

Similar to conventional cohort study which estimates the effect of baseline exposure (${x}_{0}$) on the follow-up disease endpoint (${y}_{1}$) given all participants being free of that disease at baseline (control ${y}_{0}$), the change-score analysis essentially aims to obtain the effect of ${x}_{0}$ on ${y}_{1}$ (the part that has not already been determined by ${y}_{0}$) in the setting of continuous outcomes [28]. The underlying possible linear causal model is depicted as model (3), which considers a “true state dependence” of the outcome over time (that is, the baseline outcome would causally influence the subsequent outcome).

3 $${y}_{i1}={\beta }^{{\prime }}{x}_{i0}+{\gamma }^{{\prime }}{z}_{i0}+{\rho }^{{\prime }}{y}_{i0}+{u}_{i}+{\lambda }_{0}+{\epsilon }_{i1}$$

Rather than adjusting the baseline outcome directly in the regression model, the construction of change score (${\varDelta y=y}_{1}-{y}_{0}$) likely attempts to remove the influence of baseline outcome through subtraction, and the analysis model is constructed as model (4):

4 $${y}_{i1}-{y}_{i0}={\alpha }^{{\prime }}+{\beta }^{{\prime }}{x}_{i0}+{\gamma }^{{\prime }}{z}_{i0}+{\epsilon }_{i}^{*}$$

In terms of the targeted estimand, change-score analysis aims to estimate the effect of exposure on the outcome after a one-period timespan (typically several years in cohort studies), which is the average causal effect of dairy intake many years ago on current weight in this example. As discussed in a latest article, the role of the baseline outcome variable (${y}_{0}$) is key to the success of change-score analysis, which does not provide desired causal-effect estimates (the effect of ${x}_{0}$ on ${y}_{1}$) unless the baseline outcome variable is independent of baseline exposure [28]. In addition, the change-score analysis is always biased when there exists unmeasured confounding [28].

#### Lagged change-change analysis

The lagged change-change analysis intends to guarantee the temporality of the association between exposure and outcome through a one-period lag compared with concurrent change-change analysis. This method is identical to the “lagged first-difference (LFD)” model, the linear causal model of which is depicted as model (5).

5 $${y}_{i,t+1}={\beta }^{{\prime }{\prime }}{x}_{it}+{\gamma }^{{\prime }{\prime }}{z}_{it}+{u}_{i}+{\lambda }_{t}+{\epsilon }_{i,t+1}$$

Suppose we have three-wave $t=0, 1, 2$ panel data of $i=1,\cdots ,N$ individuals, we can obtain two equations according to model (5):

$${y}_{i,1}={\beta }^{{\prime }{\prime }}{x}_{i,0}+{\gamma }^{{\prime }{\prime }}{z}_{i,0}+{u}_{i}+{\lambda }_{0}+{\epsilon }_{i1}$$

$${y}_{i,2}={\beta }^{{\prime }{\prime }}{x}_{i,1}+{\gamma }^{{\prime }{\prime }}{z}_{i,1}+{u}_{i}+{\lambda }_{1}+{\epsilon }_{i2}$$

Differencing the above two equations, we could obtain the typical analytical model used in lagged change-change analysis:

6 $${\varDelta y}_{i,(2-1)}={\alpha }^{*}+{\beta }^{{\prime }{\prime }}{\varDelta x}_{i,(1-0)}+{\gamma }^{{\prime }{\prime }}{\varDelta z}_{i,(1-0)}+{\epsilon }_{i}^{**}$$

It is not difficult to find that the lagged change-change analysis is very similar to the concurrent change-change analysis. It could also deal with the unmeasured time-invariant confounding ${u}_{i}$, except that it assumes the effect time window of exposure on outcome is one-period lagged rather than concurrent (compare the causal model (1) and (5)). Therefore, the targeted estimand of the lagged change-change analysis is the effect of exposure on outcome measured one-period later. The key to the lagged change-change analysis is the correct specification of temporal lags. The estimates would suffer from severe bias once the temporal lag does not specify the true time window of causal effects in real-world [29, 30].

A succinct comparison and summary of different analysis approaches is given in Table 1.

**Table 1:** The comparison of different analysis approaches

Approaches	Model	Estimand	Ability to solve unobserved time-invariant confounding
Concurrent change-change analysis	${\varDelta Y}_{1}=\widehat{\alpha }+\widehat{\beta }{\varDelta X}_{1}+\widehat{\gamma }{\varDelta Z}_{1}$	Concurrent effect	Yes
Change-score analysis	${\varDelta Y}_{1}={\widehat{\alpha }}^{*}+{\widehat{\beta }}^{*}{X}_{0}+{\widehat{\gamma }}^{*}{Z}_{0}$	One-period lagged effect	No
Lagged change-change analysis	$${\varDelta Y}_{2}={\widehat{\alpha }}^{**}+{\widehat{\beta }}^{**}{\varDelta X}_{1}+{\widehat{\gamma }}^{**}{\varDelta Z}_{1}$$	One-period lagged effect	Yes

#### Which estimand is more appropriate?

It is important to note that these three methods have distinct estimands, thus the choice of method should depend on which estimand best aligns with the research question. We focus on the setting of prospective studies aiming to evaluate the effect of diet on intermediate disease markers, which estimand is more appropriate in such a scenario? We think it is the concurrent change-change analysis, the reasons are as follows:

First, in a typical prospective cohort study, dietary habits in the previous year are usually retrospectively assessed using relevant questionnaires, and the biomarkers are instantly measured at each survey wave. In addition, it usually conducts repeated surveys at intervals of several years. Therefore, the concurrent effect estimand of concurrent change-change analysis corresponds to a nearly one-year effect time window, while the one-period lagged effect estimand of change-score analysis and lagged change-change analysis corresponds to the effect of diet many years ago on current intermediate disease biomarkers. Given that the intermediate disease biomarkers are usually highly sensitive and reversible (fluctuate over short periods of time as exposure changes), such concurrent effect estimand is more plausible and relevant for studying the present research question.

Second, the empirical study has shown the unbiasedness and superiority of concurrent change-change analysis, which indirectly confirms the rationality of the underlying causal model and estimand for the concurrent change-change analysis in this research question (if the estimand of the other two analysis methods captures the true causal mechanism, the empirical study would show a very different result).

#### The illustration using DAG

**Fig. 1:**
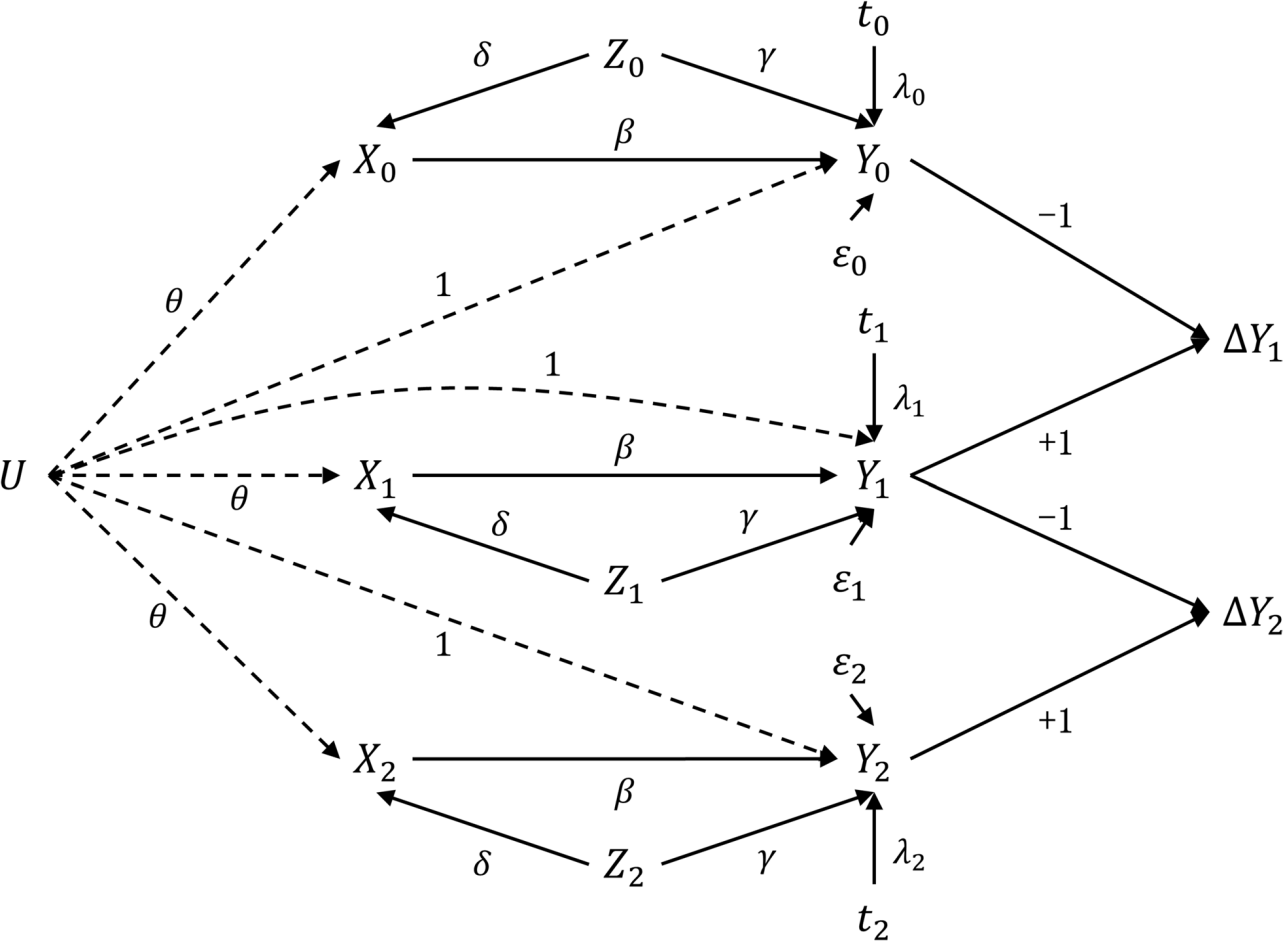
Hypothetical directed acyclic graph with three-wave panel data. $X, Y, Z$ denote the exposure, outcome, and covariate variables of interest, respectively. $U$ is the unobserved time-invariant individual characteristic. The subscripted numbers indicate the wave of the longitudinal data. The directed arrow implies the causal relation from the cause pointing to the outcome. The Greek alphabet or number on the arrow represents the causal effect of the path

DAG is a useful tool for visually displaying the causal relationships between variables and prompting how to obtain a valid causal effect based on some criteria (such as back-door criterion) [31]. Given the concurrent effect might be the most relevant and plausible estimand when studying the diet and intermediate biomarkers in prospective cohorts, we would construct a simplified DAG based on concurrent causal relation between variables, and illustrate why concurrent change-change analysis could, while change-score analysis and lagged change-change analysis could not produce a valid causal effect estimate of interest. Taking three-wave panel data for example, the constructed DAG is as in Fig. 1, and the desired estimand is $\beta$, the concurrent effect of dairy intake on weight.

#### Concurrent change-change analysis

The concurrent change-change analysis model is:

7 $${\varDelta Y}_{1}=\widehat{\alpha }+\widehat{\beta }{\varDelta X}_{1}+\widehat{\gamma }{\varDelta Z}_{1}=\widehat{\alpha }+\widehat{\beta }{(X}_{1}-{X}_{0})+\widehat{\gamma }({Z}_{1}-{Z}_{0})=\widehat{\alpha }-\widehat{\beta }{X}_{0}+\widehat{\beta }{X}_{1}-\widehat{\gamma }{Z}_{0}+\widehat{\gamma }{Z}_{1}$$

At first, we examine what is the coefficient of ${X}_{0}$ ($-\widehat{\beta }$) estimate. The true causal path of ${X}_{0}$ to $\varDelta {Y}_{1}$ is ${X}_{0}\to {Y}_{0}\to \varDelta {Y}_{1}$, and we know the true causal effect is $-\beta$ (multiplying the path coefficients in DAG), and we can find out all the noncausal paths (back-door paths) between them:

- ① $${X}_{0}\leftarrow {Z}_{0}\to {Y}_{0}\to \varDelta {Y}_{1}$$;
- ② $${X}_{0}\leftarrow U\to {Y}_{0}\to \varDelta {Y}_{1}$$;
- ③ $${X}_{0}\leftarrow U\to {Y}_{1}\to \varDelta {Y}_{1}$$;
- ④ $${X}_{0}\leftarrow U\to {X}_{1}\to {Y}_{1}\to \varDelta {Y}_{1}$$;
- ⑤ $${X}_{0}\leftarrow U\to {X}_{1}\leftarrow {Z}_{1}\to {Y}_{1}\to \varDelta {Y}_{1}$$;

The path ① is blocked by conditioning on ${Z}_{0}$; the confounding caused by the path ② and path ③ is offset due to the equivalent effect of unobserved characteristics on outcomes in each wave; the path ④ was blocked because of the inclusion of ${X}_{1}$ in the regression model; ${X}_{1}$ is a collider in the path ⑤, adjusting for ${X}_{1}$ and ${Z}_{1}$ at the same time could block the path ⑤. Thus, all the five noncausal paths have been blocked or canceled out, the coefficient $-\widehat{\beta }$in model (7) is an unbiased estimate of the causal effect of ${X}_{0}$ on $\varDelta {Y}_{1}$, that is, $-\widehat{\beta }=-\beta$. Similarly, we could also find that the coefficients of ${X}_{1}$, ${Z}_{0}$ and ${Z}_{1}$ are unbiased causal estimates for corresponding explanatory variables, thus, $\widehat{\beta }=\beta$, $\widehat{\gamma }=\gamma$. In addition, the intercept $\widehat{\alpha }$in model (7) represents the time-specific effects on $\varDelta {Y}_{1}$ (that is, ${\lambda }_{1}-{\lambda }_{0}$).

#### Change-score analysis

The change-score analysis model is:

8 $${\varDelta Y}_{1}={\widehat{\alpha }}^{*}+{\widehat{\beta }}^{*}{X}_{0}+{\widehat{\gamma }}^{*}{Z}_{0}$$

As depicted above, the true causal effect of ${X}_{0}$ on ${\varDelta Y}_{1}$ is $-\beta$. Therefore, even model (8) could yield an unbiased estimate for the causal effect of ${X}_{0}$ on ${\varDelta Y}_{1}$, which is opposite to the desired estimand $\beta .$ Furthermore, the noncausal path ④ (${X}_{0}\leftarrow U\to {X}_{1}\to {Y}_{1}\to \varDelta {Y}_{1}$) is open because ${X}_{1}$ is not adjusted in model (8), which could further introduce bias in the estimation. Thus, the estimates of change-score analysis are neither the desired estimand $\beta$ nor its opposite value.

#### Lagged change-change analysis

The lagged change-change analysis model is:

9 $${\varDelta Y}_{2}={\widehat{\alpha }}^{**}+{\widehat{\beta }}^{**}{\varDelta X}_{1}+{\widehat{\gamma }}^{**}{\varDelta Z}_{1}={\widehat{\alpha }}^{**}+{\widehat{\beta }}^{**}{(X}_{1}-{X}_{0})+{\widehat{\gamma }}^{**}{(Z}_{1}-{Z}_{0})$$

According to DAG, the true causal effect of $${X}_{0}$$ on $${\varDelta Y}_{2}$$ is zero, and the true causal effect of $${X}_{1}$$ on $${\varDelta Y}_{2}$$ is $$-\beta$$. The lagged change-change analysis model wrongly restricts the coefficients of $${X}_{0}$$ and $${X}_{1}$$ to opposite values, therefore, no matter what the estimated value of the $${\widehat{\beta }}^{**}$$ is, it cannot be correct.

### Summary

As described above, the causal model and estimand behind the concurrent change-change analysis (that is, the FEM) is more plausible than that of change-score analysis and lagged change-change analysis when studying the relationship of diet with sensitive disease biomarkers. Furthermore, the FEM has an additional powerful strength to eliminate the unmeasured time-invariant confounding and thus improve the validity of the causal estimates, which has hardly been recognized in practical studies using concurrent change-change analysis. However, the success of FEM estimation depends on two vital causal identification assumptions, which might be violated in the practical study settings and thus lead to biased estimates as well (see discussion section). We therefore must be careful when adopting the concurrent change-change analysis and interpreting the results of such method.

## Methods

### Simulation design

The previous section theoretically clarifies the underlying causal model of three common methods for studying the relationship of diet and intermediate disease markers in prospective studies, and illustrates why concurrent change-change analysis could produce unbiased results while the other two approaches could not, under the most appropriate causal model using DAG. In this section, we will conduct several simulations to intuitively display and demonstrate the strengths and limitations of recommended concurrent change-change analysis. We aim to (1) compare the unbiasedness for estimates of the concurrent change-change analysis, cross-sectional analysis, change-score analysis, and lagged change-change analysis in the settings of different extent of confounding caused by unobserved individual-specific heterogeneity; (2) investigate the performance of concurrent change-change analysis in the scenarios with varying degrees of violation of the SE assumption or the CT assumption, respectively.

### Simulation data

For the first purpose, the basic data generation model of the simulations is as follows, and the sample size and panel waves are set at 1000 and 3, respectively:

$${x}_{it}=\delta {z}_{it}+{\theta u}_{i}+{\nu }_{it}, t=\text{0,1},2; i=1,\cdots ,1000$$

$${y}_{it}=\beta {x}_{it}+\gamma {z}_{it}+{u}_{i}+{\lambda }_{t}+{\epsilon }_{it}, t=\text{0,1},2; i=1,\cdots ,1000$$

${x}_{it}$ is a continuous exposure with $\beta =1$, and ${z}_{it}$ is a continuous observed confounding covariable with $\gamma =1, \delta =0.5$, ${\lambda }_{t}$ is time-specific effect with ${\lambda }_{0}=0.5, {\lambda }_{1}=1,{\lambda }_{2}=1.5$. ${u}_{i}$ is the continuous unobserved individual heterogeneity term with effect of $\theta$ for exposure and 1 for outcome, ${\nu }_{it}$ and ${\epsilon }_{it}$ are random error terms for exposure and outcome, respectively.

We model ${z}_{it}, {u}_{i}, {\nu }_{it}$ and ${\epsilon }_{it}$ as independent standard normally distributed random variables (${z}_{it}, {u}_{i}, {\nu}_{it},{\epsilon }_{it}\sim{N}\left(\text{0,1}\right)$, that is, all of these variables have a mean of 0 and a standard deviation of 1), and then generate ${x}_{it}$ and ${y}_{it}$ according to above models and effect parameters. We set $\theta$ ranging from 0 to 1 by 0.1 intervals to represent the absence or presence of increasing degrees of unobserved confounding resulting from ${u}_{i}$.

For the second purpose, we only simulate two-wave panel data. In terms of violation of SE assumption, we only consider the situation of past outcome directly affecting current outcome for simplicity. We thus add a lagged outcome term with effect of $\rho$ in outcome model as follows:

$${x}_{it}=\delta {z}_{it}+{\theta u}_{i}+{\nu }_{it}, t=\text{0,1}; i=1,\cdots ,1000$$

$${y}_{it}=\beta {x}_{it}+\gamma {z}_{it}+\rho {y}_{i,t-1}+{u}_{i}+{\lambda }_{t}+{\epsilon }_{it}, t=\text{0,1};i=1,\cdots ,1000$$

We set $\theta =1$ and $\rho$ from 0 to 1 by 0.1 intervals to indicate the absence or presence of increasing degrees of autocorrelation of outcome. The initial outcome value ${y}_{i,-1}$ is generated from model ${y}_{i,-1}={u}_{i}+{\epsilon }_{i,-1}$, in which ${\epsilon }_{i,-1}$ are sampled from $N\left(\text{0,1}\right)$, and other parameter settings and sampling process are the same as above.

In terms of violation of CT assumption, we assume there exists unobserved time-varying confounding ${\lambda }_{it}$, that is, the time-specific effects are inconstant among individuals, the data generation model is depicted as:

$${x}_{it}=\delta {z}_{it}+{\theta u}_{i}+{\lambda }_{it}+{\nu }_{it}, t=\text{0,1}; i=1,\cdots ,1000$$

$${y}_{it}=\beta {x}_{it}+\gamma {z}_{it}+{u}_{i}+\omega {\lambda }_{it}+{\epsilon }_{it}, t=\text{0,1}; i=1,\cdots ,1000$$

We model ${\lambda }_{it}$ as a standard normal variable (${\lambda }_{it}\sim{N}\left(\text{0,1}\right)$); we also set $\theta =1$ and $\omega$ ranging from − 1 to 1 in 0.2 intervals to reflect different directions and degrees of heterogenous trends. Other parameter settings and simulating processes are identical to the above.

### Analysis models

*The concurrent change-change analysis model:*

$$\Delta{y}_{i,1-0}=\hat{\alpha}+\hat{\beta}\Delta{x}_{i,1-0}+\hat{\gamma}\Delta{z}_{i,1-0}$$

*The cross-sectional analysis model:*

$$y_{i,0}=\hat{\alpha}^{\ast\ast\ast}+\hat{\beta}^{\ast\ast\ast}x_{i,0}+\hat{\gamma}^{\ast\ast\ast}z_{i,0}$$

*The change-score analysis model:*

$$\Delta{y}_{i,1-0}=\hat{\alpha}^{\ast}+\hat{\beta}^{\ast}x_{i,0}+\hat{\gamma}^{\ast}z_{i,0}$$

*The lagged change-change analysis model:*

$$\Delta{y}_{i,2-1}=\hat{\alpha}^{\ast\ast}+\hat{\beta}^{\ast\ast}\Delta{x}_{i,1-0}+\hat{\gamma}^{\ast\ast}\Delta{z}_{i,1-0}$$

For each simulation scenario, we draw 1000 artificial sample data, produce the effect estimates using corresponding analysis methods, and compute the mean of the estimates and their standard errors. A simplified flowchart of the simulation studies is provided in Supplementary Figure S1.

## Results

**Fig. 2:**
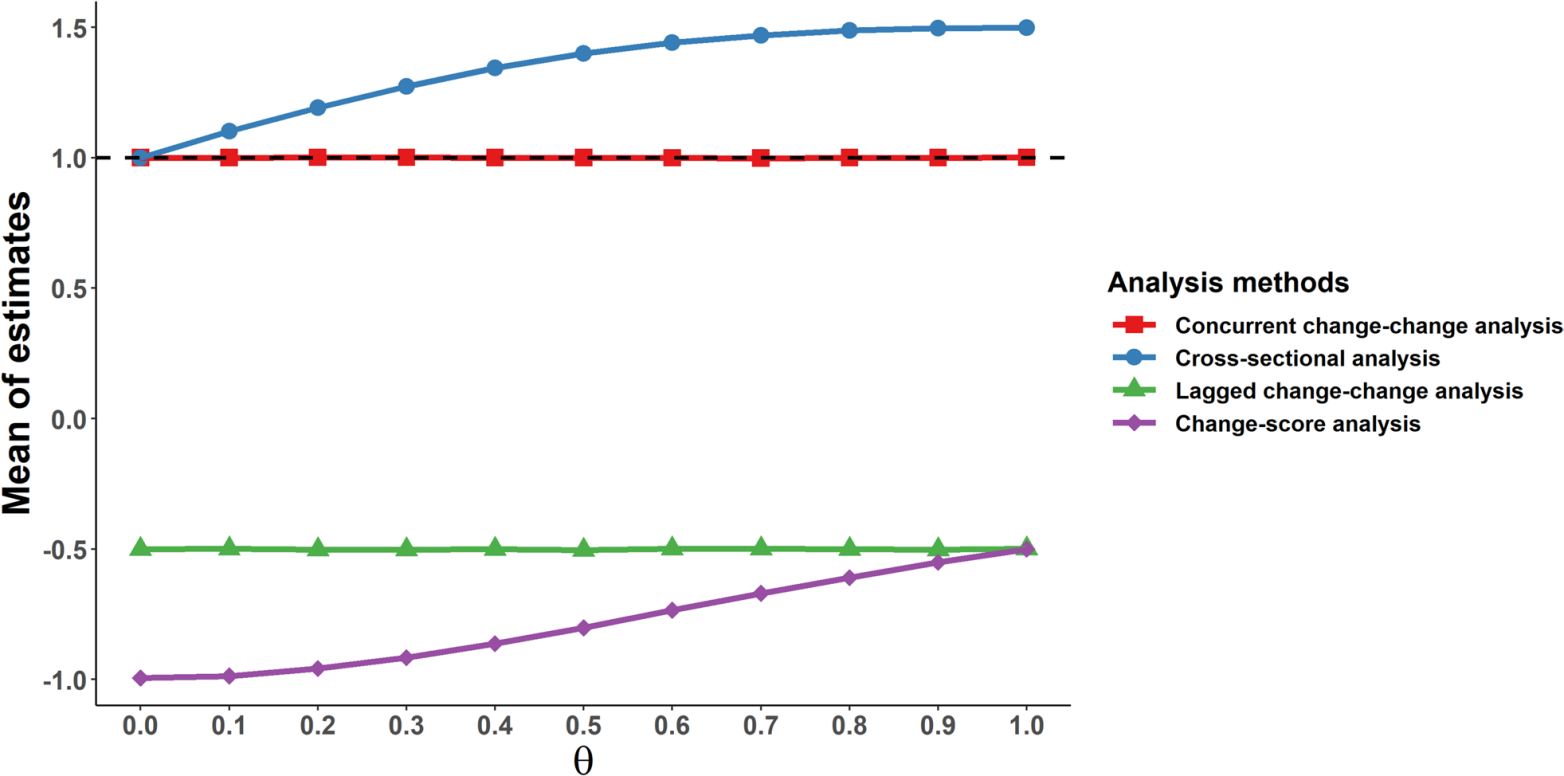
Simulation results for different change analysis models under various extents of unobserved heterogeneity. Parameter $\theta$ indicates the relation of the unobserved time-invariant individual characteristic and exposure, thus representing the extent of the unobserved confounding. The black dashed line represents the true causal effect of exposure on outcome

The trend and the estimated coefficient using different analysis methods under various degrees of unobserved heterogeneity are given in Fig. 2. Concurrent change-change analysis has always yielded unbiased estimates as expected. The cross-sectional analysis produces unbiased estimates only when there is no unobserved confounding, and produces increasingly biased estimates as the unobserved heterogeneity is larger (ranging from 0.999 to 1.499). However, the estimates of change-score analysis (ranging from − 0.995 to -0.500) and lagged change-change analysis (remains around − 0.5) are always biased and in the opposite direction of the true causal effect even without any unobserved heterogeneity. (See the Supplementary Table S1 for full details.)

**Fig. 3:**
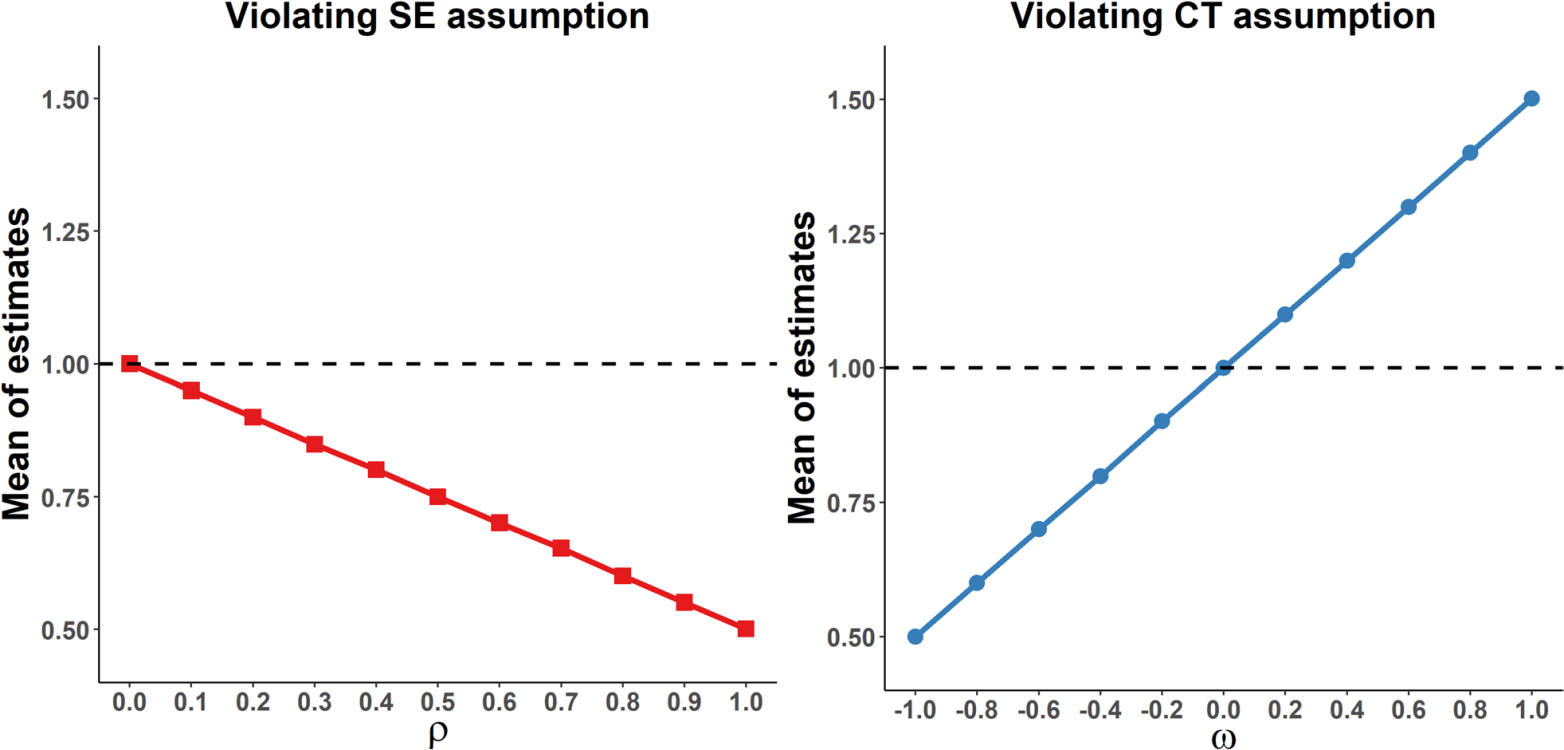
Simulation results for concurrent change-change analysis with varying degrees of violation of the strict exogeneity assumption or the common trend assumption. Parameter $\rho$ indicates the effect of the past outcome on the current outcome, thus representing the extent of violation of the strict exogeneity assumption. Parameter $\omega$ indicates the effect of unobserved time-varying confounding, thus representing the extent of violation of the common trend assumption. The black dashed line represents the true causal effect of exposure on outcome

The trend and the mean of the estimates for concurrent change-change analysis under different degrees of violation of the SE or the CT assumption are shown in Fig. 3. The results show a linear tendency of increased estimation bias as the degrees of violation become more serious (ranging from 1.000 to 0.501 for violated SE assumption, and increasing from 0.500 to 1.501 for violated CT assumption), with unbiased estimates under no violation of assumptions. (See the Supplementary Table S2 for more details.)

## Discussion

### Overview

This study thoroughly explores and understands three analysis approaches evaluating diet and intermediate disease markers in prospective studies within the causal inference framework, and mainly demonstrates the strengths and pitfalls of the concurrent change-change analysis recommended in the applied researches through simulations. We find that the underlying causal model and targeted estimand are different for distinct analysis methods. Specifically, the concurrent change-change analysis concerns the contemporaneous effect of exposure on outcome, while the change-score analysis and lagged change-change analysis target the effect of exposure on outcome after a one-period timespan. In the setting of prospective cohorts with repeated measures at several-year intervals, estimating the concurrent effect of diet on sensitive biomarkers (corresponding to a nearly one-year effect window) is more relevant and plausible in practice, and corresponding concurrent change-change analysis could yield robust and unbiased estimates even with serious unobserved time-invariant confounding. Nevertheless, the SE and CT assumptions are prerequisites for applying concurrent change-change analysis, violation of which would lead to biased results as well.

### Rationality and strength of concurrent change-change analysis

Given the targeted estimand and implied causal model are distinct for these three analysis methods, the fundamental criterion for judging the applicability of a method is which estimand is most relevant to the specific research question. As mentioned above, we think concurrent change-change analysis targets the most proper estimand in the setting of prospective studies aiming to evaluate the effect of diet on intermediate disease markers. The sensitivity and reversibility feature of intermediate biomarkers implies that the effect of exposure would generally occur within a short period of time, in other words, the recent exposures are much more important than the distant past exposures. In many randomized controlled trials (RCTs) evaluating the performance of diet or physical activity interventions on weight loss or improvement of cardiometabolic markers, the intervention time is generally several weeks to two years, within which researchers often observe significant favorable effects [32–35]. However, regain of weight or those biomarkers usually occurs within a longer follow-up period after the end of intervention [36, 37]. This phenomenon coincides with the above viewpoint and potentially reinforces the rationality of concurrent effect assumption. In addition to a more plausible estimand, the concurrent change-change analysis could circumvent the unobserved time-invariant (or relatively stable in the short term) confounding problem plaguing observational studies, such as personality, genetic susceptibility, and cultural customs [38].

### Pitfalls and relevant progress of concurrent change-change analysis (FEM)

Is concurrent change-change analysis the panacea for solving the research question about the relation between diet and disease biomarkers, given its preferable performance in empirical studies and more plausible causal model in theory? The answer is of course no. From the perspective of FEM, the use of concurrent change-change analysis is conditioned on two vital identification assumptions, which might be violated in practical research scenarios. For example, the SE assumption requires the past outcome does not directly affect the subsequent outcome, thus attributing the correlation in outcome over time to the stable unobserved individual-specific heterogeneity ${u}_{i}$ or the temporal correlation of other influencing factors (${x}_{it}$ or ${z}_{it}$) of the outcome [26]. This may be correct for many sensitive and reversible biomarkers, but not for others which usually indicate irreversible organic/pathological changes (for instance, extreme glucose metabolism indicators can reflect islet damage [39]). In above situation, the past biomarker does causally affect the later biomarker level, thus violating the SE assumption. As for the CT assumption, it requires complete homogeneity for unmeasured time-varying variables among individuals, however, many ubiquitous unmeasured time-varying health determinants such as health awareness and behavioral predisposition tend to have strong individual heterogeneity. Fortunately, there has been some methodological progress to relax the SE or CT assumption in above situations [26, 40–42]. The most classical method to loosen the SE assumption is to add the lagged dependent variable term ${y}_{i,t-1}$ into the traditional FEM model (called Dynamic Fixed Effects Model, DFEM) and combine the instrumental variables methods and generalized method of moments procedure to obtain the estimates based on first-differenced data [41]. The most simple and common method to loosen the CT assumption is to construct the fixed-effects model with individual-specific constants and slopes (FEIS) and estimate it through second differencing, thus allowing time-specific effects or the unobserved time-varying variables heterogeneous [26].

In addition to SE and CT assumptions, there are two other potential limitations for concurrent change-change analysis worthy of note. First, such within-individual estimators would lose information and lead to a lack of precision (low statistical power), thus might require a larger sample, more waves of data, and sufficient variation over time in the exposure [38, 43, 44]. Second, this method is unable to deal with the problem of reverse causality. On the one hand, modeling the (cross-sectional) relationship of concurrent exposure and outcome could not clarify the causal order, but the proper temporality could be guaranteed by the data collection method and process (retrospectively collect the exposure). On the other hand, if the reverse causation of previous outcome and current exposure exist (especially for those biomarkers that are known to or monitored by the study participants, for example, deterioration of blood glucose could cause individuals to modify their future lifestyles), concurrent change-change analysis would also yield biased estimates. Other methods such as cross-lagged panel model with fixed effects might be useful in such situations [29].

### Recommendations

1. How to choose the appropriate analysis method, and when could we adopt the concurrent change-change analysis?

Several key factors should be considered when choosing among these methods. Firstly, the research question nature and the true temporal relationship between concerned variables is the fundamental criterion, we should employ the concurrent change-change analysis when focus on immediate or short-term effect, and would prefer the change-score analysis or lagged change-change analysis when aiming to estimate the delayed or lagged effect. Secondly, we should contemplate whether there are important unobservable individual or group-specific effects that lead to confounding, if there exists such unobserved heterogeneity, the change-score analysis is not a useful method, while the other two methods can deal with such problem. Finally, the autocorrelation and serial dependency is another key point, which is considered in the change-score analysis while is not allowed in the other two methods. In conclusion, we should make the choice carefully according to different scenarios and correctly interpret the results of different methods.

Our study focuses on a specific scenario of prospective studies that seek to estimate the causal relation between diet and sensitive intermediate disease biomarkers, in which the most or all of the effect of exposure will occur within a short time. Moreover, there should exist neither dynamic causal relationships between exposure and outcome across different periods (such as “true state dependence” for the outcome indicators over time) nor clear unobserved time-varying heterogeneity in specific research questions. If so, concurrent change-change analysis is most relevant and would lead to the most robust and biologically plausible results comparable to RCTs.

(2) How to conduct the concurrent change-change analysis?

To reiterate, careful and stringent examination of the applicability for the SE and CT assumptions is necessary, if the specific research scenario substantially diverges from these assumptions, DFEM or FEIS models with corresponding estimation methods might be alternative solutions. When the concurrent change-change analysis is appropriate to conduct, we could directly model the association of change in exposure and parallel change in outcome indicator, and simultaneously adjusting for the changes in those observed time-varying confounders, with no need to include any time-invariant covariates (because both unobserved and observed time-invariant terms will be counteracted given the effects of these variables are constant over time) or the baseline level of confounders or outcomes (because when the SE assumption is satisfied, the previous outcome is not a cause for the later outcome, although adjustment for previous/baseline outcome is quite common in applied longitudinal studies).

### Strengths and weaknesses

To the best of our knowledge, this is the first study to thoroughly dissect three commonly used analysis approaches for diet and intermediate disease markers in prospective researches from the causal inference perspective, and confirms the superiority of recommended concurrent change-change analysis in theory and in simulation, which is conducive to the scientific application of these methods and improvement of the research quality. However, there are still several limitations or caveats worthy of notice. First, this study only concerns and interprets three mainstream methods, and there might be other analysis approaches in similar applied studies not considered. Second, we generate simulated data only based on the causal model of FEM and do not consider that of other analysis approaches, because empirical and theoretical evidence has suggested that it is most plausible for the research questions we care about. Third, the simulations in present study are oversimple. We did not use a specific illustrative research question and did not set the effect parameters and variable distributions according to empirical data, which might make it difficult to relate the simulation to reality. However, we mainly aimed to intuitively display the fact that concurrent change-change analysis generally outperforms other methods but returns biased estimates when the vital assumptions are violated. The magnitude of the bias resulting from improper analysis method or violation of model identification assumptions in specific research scenario is out of the scope of this article.

## Conclusions

In conclusion, the commonly used change-score analysis, concurrent change-change analysis and lagged change-change analysis target different estimands with different interpretations. Concurrent change-change analysis might be the most superior method in studying the causal relation of diet and intermediate biomarkers, which targets the most plausible estimand and tremendously ameliorates the intractable bias from unobserved individual heterogeneity in observational studies. Although this method is highly recommended, the vital assumptions behind it should be always kept in mind.

### Supplementary Information

**Additional file 1: Figure S1.** The simplified flowchart of the simulation studies. **Table S1.** The mean of estimates and standard errors using different analysis methods under different degrees of unobserved heterogeneity^a^. **Table S2.** The mean of estimates and standard errors using concurrent change-change analysis under different degrees of violation of the strict exogeneity assumption or the common trend assumption^a^.

## Data Availability

All data generated or analysed during this study are included in this published article [and its supplementary information files].

## Abbreviations

CT: Common trend
DAG: Directed acyclic graph
DFEM: Dynamic fixed effects model
FEIS: Fixed-effects model with individual-specific constants and slopes
FEM: Fixed effects model
FFQ: Food frequency questionnaire
SE: Strict exogeneity
