# Supplementary material for: Change analysis for intermediate disease markers in nutritional epidemiology: a causal inference perspective
Source: BMC Med Res Methodol. 2024 Feb 27;24:49. doi: 10.1186/s12874-024-02167-9 (PMC10898035; doi:10.1186/s12874-024-02167-9)
Supplement: Supplementary file 1 — Additional file 1: Figure S1. The simplified flowchart of the simulation studies. Table S1. The mean of estimates and standard errors using different analysis methods under different degrees of unobserved heterogeneitya. Table S2. The mean of estimates and standard errors using concurrent change-change analysis under different degrees of violation of the strict exogeneity assumption or the common trend assumptiona. [file 12874_2024_2167_MOESM1_ESM.docx]

**Online Supplementary Material**

**Article title**

Change analysis for intermediate disease markers in nutritional epidemiology: a causal inference perspective

**Journal**

BMC Medical Research Methodology

**Authors**

Dan Tang^1,2^, Yifan Hu^1^, Ning Zhang^1^, Xiong Xiao^1*^, Xing Zhao^1*^

^1^ West China School of Public Health and West China Fourth Hospital, Sichuan University, Chengdu, China;

^2^ Xiamen Center for Disease Control and Prevention, Xiamen, China;

***Correspondence:**

Prof. Xing Zhao, Department of Epidemiology and Biostatistics, West China School of Public Health and West China Fourth Hospital, Sichuan University, Chengdu, Sichuan, CN 610041. Email: [xingzhao@scu.edu.cn](mailto:xingzhao@scu.edu.cn). Tel: +86-132-5818-3126

OR

Dr. Xiong Xiao, Department of Epidemiology and Biostatistics, West China School of Public Health and West China Fourth Hospital, Sichuan University, Chengdu, Sichuan, CN 610041. Email: [xiaoxiong.scu@scu.edu.cn](mailto:xiaoxiong.scu@scu.edu.cn). Tel: +86-134-3808-8578


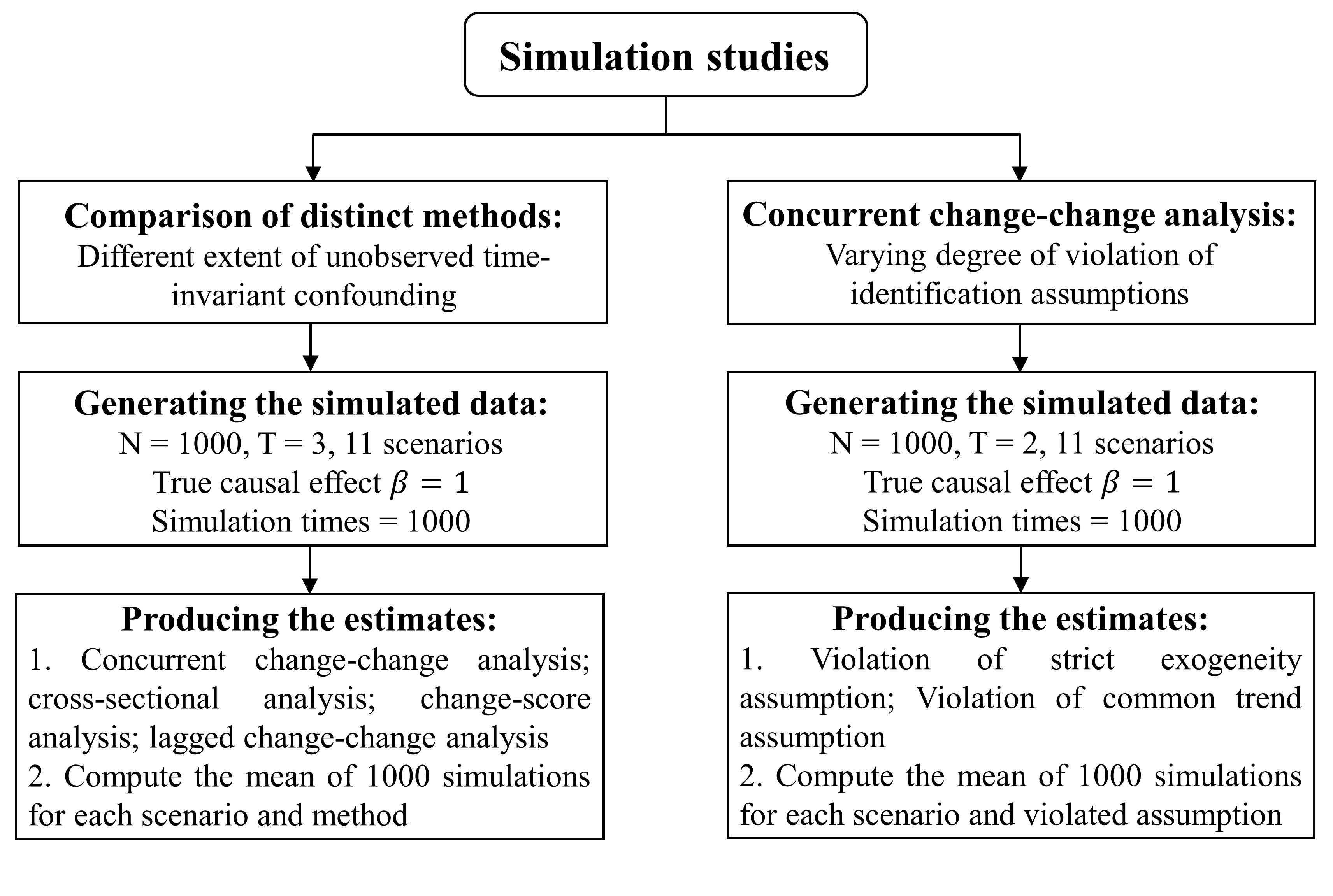


Figure S1. The simplified flowchart of the simulation studies.

Table S1. The mean of estimates and standard errors using different analysis methods under different degrees of unobserved heterogeneity ^a^.

| **Parameter** $\boldsymbol{\theta}$**^b^** | **Analysis methods** | | | |
| --- | --- | --- | --- | --- |
|  | Concurrent change-change analysis | Cross-sectional analysis | Change-score analysis | Lagged change-change analysis |
| **0** | 1.000 (0.032) | 0.999 (0.045) | -0.995 (0.073) | -0.501 (0.059) |
| **0.1** | 1.000 (0.032) | 1.101 (0.045) | -0.987 (0.072) | -0.499 (0.059) |
| **0.2** | 1.001 (0.032) | 1.191 (0.044) | -0.958 (0.072) | -0.502 (0.059) |
| **0.3** | 1.001 (0.032) | 1.273 (0.042) | -0.917 (0.070) | -0.503 (0.059) |
| **0.4** | 1.000 (0.032) | 1.345 (0.040) | -0.863 (0.068) | -0.500 (0.059) |
| **0.5** | 1.000 (0.032) | 1.399 (0.038) | -0.803 (0.066) | -0.504 (0.059) |
| **0.6** | 0.999 (0.032) | 1.441 (0.036) | -0.734 (0.064) | -0.498 (0.059) |
| **0.7** | 0.998 (0.032) | 1.470 (0.034) | -0.670 (0.061) | -0.499 (0.059) |
| **0.8** | 1.000 (0.032) | 1.488 (0.031) | -0.610 (0.059) | -0.500 (0.059) |
| **0.9** | 1.000 (0.032) | 1.497 (0.029) | -0.551 (0.056) | -0.502 (0.059) |
| **1.0** | 1.001 (0.032) | 1.499 (0.027) | -0.500 (0.054) | -0.499 (0.059) |

a. For each scenario of different extents of unobserved heterogeneity, 1000 simulations are conducted, and the mean of the results are presented.

b. $\theta$ is the parameter indicating the relation of the unobserved time-invariant individual characteristic and exposure, thus representing the extent of the unobserved confounding.

Table S2. The mean of estimates and standard errors using concurrent change-change analysis under different degrees of violation of the strict exogeneity assumption or the common trend assumption ^a^.

| **Violation of SE assumption** | |  | **Violation of CT assumption** | |
| --- | --- | --- | --- | --- |
| **Parameter** $\boldsymbol{\rho}$**^b^** | Estimates (se) |  | **Parameter** $\boldsymbol{\omega}$**^c^** | Estimates (se) |
| **0** | 1.000 (0.032) |  | **-1.0** | 0.500 (0.027) |
| **0.1** | 0.950 (0.030) |  | **-0.8** | 0.600 (0.026) |
| **0.2** | 0.900 (0.030) |  | **-0.6** | 0.700 (0.024) |
| **0.3** | 0.849 (0.030) |  | **-0.4** | 0.799 (0.023) |
| **0.4** | 0.801 (0.032) |  | **-0.2** | 0.901 (0.023) |
| **0.5** | 0.750 (0.034) |  | **0** | 1.000 (0.022) |
| **0.6** | 0.701 (0.037) |  | **0.2** | 1.099 (0.023) |
| **0.7** | 0.653 (0.041) |  | **0.3** | 1.199 (0.023) |
| **0.8** | 0.601 (0.046) |  | **0.6** | 1.299 (0.024) |
| **0.9** | 0.551 (0.051) |  | **0.8** | 1.400 (0.026) |
| **1.0** | 0.501 (0.058) |  | **1.0** | 1.501 (0.027) |

a. For each scenario of different extents of violation for identification assumptions, 1000 simulations are conducted, and the mean of the results are presented.

b. $\rho$ is the parameter indicating the autocorrelation of the past outcome and the current outcome, thus representing the extent of violation of the strict exogeneity assumption.

c. $\omega$ is the parameter indicating the effect of the unobserved time-varying confounder, thus representing the extent of violation of the common trend assumption.

**Simulation code**

library(tidyverse)

library(MASS)

### simulation 1：compare different methods under varying extent of individual-specific heterogeneity####-----------------

### generate simulation data 1：

sim.times <- 1000

THETA <- seq(from = 0, to = 1, by = 0.1)### Parameter θ indicates the relation of the unobserved time-invariant individual characteristic and exposure

n <- length(THETA)

### create an empty table to store the results

result <- data.frame(theta = rep(THETA,each = 4*sim.times),

Analysis_methods = rep(rep(c("Concurrent change-change analysis","Cross-sectional analysis","Change-score analysis","Lagged change-change analysis"),each = sim.times),n),

sim_num = rep(1:sim.times,4*n),estimate = NA, se = NA)

### run the Loop

set.seed(100)

for (s in 1:n) {

theta <- THETA[s] #### varying individual-specific heterogeneity

for (sim in 1:sim.times) {

### Generate covariate and individual-specific item

### three waves of covariate data

mean <- c(0,0,0)

sigma <- matrix(c(1,0,0,0,1,0,0,0,1),nrow=3,ncol=3)### Independent normal distribution

z_i <- as.data.frame(mvrnorm(n=1000,mean,sigma))

colnames(z_i) <- c("z_i0","z_i1","z_i2")

### individual-specific item

u_i <- rnorm(1000,mean = 0,sd = 1)

### random error items for exposure and outcome

niu_i <- as.data.frame(mvrnorm(n=1000,mean,sigma))

epsilon_i <- as.data.frame(mvrnorm(n=1000,mean,sigma))

colnames(niu_i) <- c("niu_i0","niu_i1","niu_i2")

colnames(epsilon_i) <- c("epsilon_i0","epsilon_i1","epsilon_i2")

data <- cbind(z_i,niu_i,epsilon_i,u_i)

### define the effect parameter

lambda_0 <- 0.5; lambda_1 <- 1; lambda_2 <- 1.5

beta <- 1; gamma <- 1; delta <- 0.5

### Generate exposure data

x_i0 <- delta*data$z_i0 + theta*data$u_i + data$niu_i0

x_i1 <- delta*data$z_i1 + theta*data$u_i + data$niu_i1

x_i2 <- delta*data$z_i2 + theta*data$u_i + data$niu_i2

### Generate outcome data

y_i0 <- beta*x_i0 + gamma*data$z_i0 + data$u_i + lambda_0 + data$epsilon_i0

y_i1 <- beta*x_i1 + gamma*data$z_i1 + data$u_i + lambda_1 + data$epsilon_i1

y_i2 <- beta*x_i2 + gamma*data$z_i2 + data$u_i + lambda_2 + data$epsilon_i2

### Synthesize data

df_FEM <- cbind(data,x_i0,x_i1,x_i2,y_i0,y_i1,y_i2)%>%

mutate(Δy_i1 = y_i1 - y_i0, Δx_i1 = x_i1 - x_i0, Δz_i1 = z_i1 - z_i0,

Δy_i2 = y_i2 - y_i1, Δx_i2 = x_i2 - x_i1, Δz_i2 = z_i2 - z_i1)

### the list of model formulas

formula <- vector("list",4)

formula[[1]] <- Δy_i1 ~ Δx_i1 + Δz_i1 ## concurrent change-change analysis

formula[[2]] <- y_i0 ~ x_i0 + z_i0 ## cross-sectional analysis

formula[[3]] <- Δy_i1 ~ x_i0 + z_i0 ## change-score analysis

formula[[4]] <- Δy_i2 ~ Δx_i1 + Δz_i1 ## lagged change-change analysis

for (j in 1:4) {

model <- coef(summary(lm(formula[[j]], data = df_FEM)))

result[sim + sim.times*(j-1) + 4*sim.times*(s-1),4] <- model[2,1]

result[sim + sim.times*(j-1) + 4*sim.times*(s-1),5] <- model[2,2]

}

}

}

### Summary of the results

simulate_1 <- result %>%

group_by(theta, Analysis_methods) %>%

summarise(est_mean = round(mean(estimate),3),

se_mean = round(mean(se),3))

save(simulate_1,file = "simulation1 results.Rdata")

### simulation 2：the bias of concurrent change-change analysis with varying degrees of violation of the SE assumption####-----------------

### add a lagged outcome item

sim.times <- 1000

RHO <- seq(from = 0, to = 1, by = 0.1)### autocorrelation coefficient of the outcome

n <- length(RHO)

### create an empty table to store the results

result <- data.frame(rho = rep(RHO,each = sim.times), Violated_assumption = "SE assumption",

sim_num = rep(1:sim.times,n),estimate = NA, se = NA)

### run the Loop

set.seed(100)

for (s in 1:n) {

rho <- RHO[s] #### varying autocorrelation

for (sim in 1:sim.times) {

### Generate covariate and individual-specific item

### two waves of covariate data

mean <- c(0,0)

sigma <- matrix(c(1,0,0,1),nrow=2,ncol=2)

z_i <- as.data.frame(mvrnorm(n=1000,mean,sigma))

colnames(z_i) <- c("z_i0","z_i1")

### individual-specific item

u_i <- rnorm(1000,mean = 0,sd = 1)

### random error items for exposure and outcome

niu_i <- as.data.frame(mvrnorm(n=1000,mean,sigma))

epsilon_i <- as.data.frame(mvrnorm(n=1000,mean,sigma))

colnames(niu_i) <- c("niu_i0","niu_i1")

colnames(epsilon_i) <- c("epsilon_i0","epsilon_i1")

### Generate the initial outcome value y_(i,-1)

y_ii <- u_i + rnorm(n=1000,mean=0,sd=1)

data <- cbind(z_i,niu_i,epsilon_i,u_i,y_ii)

### define the effect parameter

lambda_0 <- 0.5; lambda_1 <- 1;

beta <- 1; gamma <- 1; delta <- 0.5; theta <- 1

### Generate exposure data

x_i0 <- delta*data$z_i0 + theta*data$u_i + data$niu_i0

x_i1 <- delta*data$z_i1 + theta*data$u_i + data$niu_i1

### Generate outcome data

y_i0 <- beta*x_i0 + gamma*data$z_i0 + rho*data$y_ii + data$u_i + lambda_0 + data$epsilon_i0

y_i1 <- beta*x_i1 + gamma*data$z_i1 + rho*y_i0 + data$u_i + lambda_1 + data$epsilon_i1

### Synthesize data

df_FEM <- cbind(data,x_i0,x_i1,y_i0,y_i1)%>%

mutate(Δy_i1 = y_i1 - y_i0, Δx_i1 = x_i1 - x_i0, Δz_i1 = z_i1 - z_i0)

### run the model

model <- coef(summary(lm(Δy_i1 ~ Δx_i1 + Δz_i1, data = df_FEM)))

result[sim + sim.times*(s-1),4] <- model[2,1]

result[sim + sim.times*(s-1),5] <- model[2,2]

}

}

### Summary of the results

simulate_2 <- result %>%

group_by(rho, Violated_assumption) %>%

summarise(est_mean = round(mean(estimate),3),

se_mean = round(mean(se),3))

save(simulate_2,file = "simulation2 results.Rdata")

### simulation 3：the bias of concurrent change-change analysis with varying degrees of violation of the CT assumption####-----------------

### add an unobserved time-varying confounding λ_it,

sim.times <- 1000

OMEGA <- seq(from = -1, to = 1, by = 0.2)### Parameter ω indicates the relation of unobserved time-varying confounder and outcome

n <- length(OMEGA)

### create an empty table to store the results

result <- data.frame(omega = rep(OMEGA,each = sim.times), Violated_assumption = "CT assumption",

sim_num = rep(1:sim.times,n),estimate = NA, se = NA)

### run the Loop

set.seed(100)

for (s in 1:n) {

omega <- OMEGA[s] #### varying unobserved time-varying confounding

for (sim in 1:sim.times) {

### Generate covariate and individual-specific item

### two waves of covariate data

mean <- c(0,0)

sigma <- matrix(c(1,0,0,1),nrow=2,ncol=2)

z_i <- as.data.frame(mvrnorm(n=1000,mean,sigma))

colnames(z_i) <- c("z_i0","z_i1")

### individual-specific item

u_i <- rnorm(1000,mean = 0,sd = 1)

### random error items for exposure and outcome

niu_i <- as.data.frame(mvrnorm(n=1000,mean,sigma))

epsilon_i <- as.data.frame(mvrnorm(n=1000,mean,sigma))

colnames(niu_i) <- c("niu_i0","niu_i1")

colnames(epsilon_i) <- c("epsilon_i0","epsilon_i1")

### unobserved time-varying confounder item

lambda_i <- as.data.frame(mvrnorm(n=1000,mean,sigma))

colnames(lambda_i) <- c("lambda_i0","lambda_i1")

data <- cbind(z_i,niu_i,epsilon_i,u_i,lambda_i)

### define the effect parameter

beta <- 1; gamma <- 1; delta <- 0.5; theta <- 1

### Generate exposure data

x_i0 <- delta*data$z_i0 + theta*data$u_i + data$lambda_i0 + data$niu_i0

x_i1 <- delta*data$z_i1 + theta*data$u_i + data$lambda_i1 + data$niu_i1

### Generate outcome data

y_i0 <- beta*x_i0 + gamma*data$z_i0 + data$u_i + omega*data$lambda_i0 + data$epsilon_i0

y_i1 <- beta*x_i1 + gamma*data$z_i1 + data$u_i + omega*data$lambda_i1 + data$epsilon_i1

### Synthesize data

df_FEM <- cbind(data,x_i0,x_i1,y_i0,y_i1)%>%

mutate(Δy_i1 = y_i1 - y_i0, Δx_i1 = x_i1 - x_i0, Δz_i1 = z_i1 - z_i0)

### run the model

model <- coef(summary(lm(Δy_i1 ~ Δx_i1 + Δz_i1, data = df_FEM)))

result[sim + sim.times*(s-1),4] <- model[2,1]

result[sim + sim.times*(s-1),5] <- model[2,2]

}

}

### Summary of the results

simulate_3 <- result %>%

group_by(omega, Violated_assumption) %>%

summarise(est_mean = round(mean(estimate),3),

se_mean = round(mean(se),3))

save(simulate_3,file = "simulation3 results.Rdata")
